# Supplementary material for: Rapeseed Protein Isolate as a Sustainable Alternative to Soy Protein: A Case Study on Chicken Pâtés
Source: Foods. 2025 Nov 10;14(22):3841. doi: 10.3390/foods14223841 (PMC12651233; doi:10.3390/foods14223841)
Supplement: Supplementary file 1 [file foods-14-03841-s001.zip › foods-3933088-supplementary.pdf]

Supplementary Table S1. Amino acid composition of rapeseed protein isolate (RPI) and soybean protein isolate (SPI) used in formulation of chicken pâtés.

**Amino acid composition (g/100 sample)**	**Rapeseed protein isolate (RPI)***	**Soy protein isolate (SPI)****
Aspartic acid	6.33 ± 0.07	10.7
Threonine	3.42 ± 0.05	3.20
Serine	3.71 ± 0.04	5.00
Glutamic acid	11.3 ± 0.08	18.0
Proline	8.30 ± 0.16	4.70
Glycine	3.41 ± 0.01	3.70
Alanine	3.32 ± 0.01	3.70
Cysteine	-	1.20
Valine	3.89 ± 0.04	4.00
Methionine	1.32 ± 0.29	1.20
Isoleucine	4.27 ± 0.02	3.80
Leucine	7.28 ± 0.05	6.90
Tyrosine	2.11 ± 0.05	3.60
Phenylalanine	3.07 ± 0.03	4.80
Histidine	3.62 ± 0.10	2.40
Lysine	5.31 ± 0.15	5.70
Arginine	8.00 ± 0.13	7.10
Total	78.6 ± 0.57	89.7

* own data; ** manufacturer's specification

Supplementary Table S2. Fatty-acid composition of rapeseed oil and sunflower oil used in formulation of chicken pâtés (F1-F4).

Fatty-acid composition (g/100g oil)	Rapeseed oil*		Sunflower oil*	
**C14:0**	0.07	± 0.00	0.06	± 0.00
**C16:0**	5.29	± 0.13	6.43	± 0.02
**C16:1**	0.46	± 0.01	0.12	± 0.01
**C17:0**	-	-	0.04	± 0.00
**C17:1**	-	-	0.04	± 0.00
**C18:0**	1.49	± 0.04	4.03	± 0.04
**C18:1 n-9**	60.6	± 2.08	27.1	± 0.23
**C18:2 n-6**	20.9	± 0.62	60.4	± 0.19
**C18:3 n-3**	9.44	± 1.23	0.12	± 0.00
**C20:0**	0.55	± 0.05	0.72	± 0.08
**C20:1 n-9**	-	-	0.12	± 0.02
**C20:2n-6**	0.10	± 0.00	-	-
**C22:0**	0.32	± 0.05	0.71	± 0.03
**C22:2n-6**	0.20	± 0.01	-	-
**C20:3 n-3**	-	-	0.05	± 0.04
**C24:0**	0.16	± 0.02	-	-
**∑ SFA**	7.87	± 0.10	12.0	± 0.10
**∑ MUFA**	61.1	± 0.27	27.4	± 0.27
**∑ PUFA**	30.7	± 0.16	60.6	± 0.16

* own data;

SFA - saturated fatty acids; MUFA - monounsaturated fatty acids; PUFA - polyunsaturated fatty acids

Supplementary Table S3. Proximate composition of chicken meat and liver used in formulation of chicken pâtés.

Proximates (g/100 g)	Chicken breast*	Chicken thigh*	Chicken drumstick*	Chicken liver*
**Moisture**	74.8	72.9	75.3	76.5
**Ash**	1.13	0.96	0.89	1.06
**Fat**	1.93	7.92	5.94	4.83
**Protein**	22.5	18.6	18.4	16.9
**Carbohydrates**	0	0	0	0.73

* USDA - FoodData Central
